# Novel recombinant protein flagellin A N/C attenuates experimental autoimmune encephalomyelitis by suppressing the ROS/NF-κB/NLRP3 signaling pathway

**DOI:** 10.3389/fphar.2022.956402

**Published:** 2022-11-14

**Authors:** Li Li, Shihua Deng, Mingquan Liu, Min Yang, Jin Li, Teng Liu, Ting Zhang, Yangyang Zhao, Miao He, Dongming Wu, Ying Xu

**Affiliations:** ^1^ Clinical Medical College and the First Affiliated Hospital of Chengdu Medical College, Chengdu, China; ^2^ Sichuan Clinical Research Center for Geriatrics, The First Affiliated Hospital, Chengdu Medical College, Chengdu, China

**Keywords:** FlaAN/C, experimental autoimmune encephalomyelitis, reactive oxygen species, ROS/NF-κB/NLRP3 pathway, pyroptosis

## Abstract

Multiple sclerosis (MS) is a chronic inflammatory autoimmune disease characterized by demyelination and neurodegeneration, for which traditional treatment offers limited relief. Microglial/macrophage modulation plays a critical role in the pathogenesis of MS. Oxygen free radical accumulation can induce axonal and nerve cell damage, and further promote MS development. We created a new recombinant protein based on flagellin from *Legionella pneumophila* named flagellin A with linked *C*- and *N*-terminal ends (FLaAN/C), which is an independent intellectual property of our team. We previously showed that FLaAN/C might mitigate radiation-induced damage by inhibiting inflammatory responses and oxidative stress. However, whether FLaAN/C protects against MS remains unknown. Here, we investigated the anti-inflammatory effects of FLaAN/C on mice with experimental autoimmune encephalomyelitis (EAE) induced by oligodendrocyte glycoprotein peptide 35–55 (MOG35-55). The mice were injected intraperitoneally with FLaAN/C after the onset of clinical symptoms, then clinical behavior scores and changes in body weight were recorded daily. The spinal lumbar spine in model mice was enlarged and accompanied by inflammatory cell infiltration and demyelination that were reversed by FLaAN/C. FLaAN/C also induced microglia/macrophages to generate less pro-inflammatory (CD86, iNOS, and TNF-α), and more anti-inflammatory (CD206, IL-10, and Arginase-1) cytokines. These findings suggesting that FLaAN/C promoted microglial/macrophages polarization from the inflammatory M1 to the anti-inflammatory M2 phenotype. Moreover, FLaAN/C inhibited release of the inflammatory cytokines, TNF-α, IL-8, IL-6, IL-17, and IFN-γ. These results indicated that the anti-inflammatory effect of FLaAN/C was associated with the inhibited generation of reactive oxygen species. FLaAN/C downregulated the expression of phosphorylated NF-κB-p65 and prevented downstream NLRP3 inflammasome-mediated pyroptosis. Collectively, these results indicated that FLaAN/C prevents pyroptosis by inhibiting the ROS/NF-κB/NLRP3 signaling pathway, and promotes the microglial/macrophage M1/M2 polarization that significantly alleviated inflammation in mouse models of EAE. Our findings suggested that FLaAN/C could be a promising candidate for MS therapy.

## Introduction

Multiple sclerosis (MS) is a chronic inflammatory autoimmune disease of the central nervous system (CNS) that is characterized by multiple lesions, remission, recurrence, and other features (Yong, 2022). The main pathologies are inflammatory cell infiltration, axon loss or damage, white matter demyelination, and chronic neuroinflammation (Correale et al., 2017). Multiple sclerosis is more prevalent in younger individuals, but often continues to progress to advanced stages, leading to severe neurological deficits and loss of the ability to work (Dobson and Giovannoni, 2019). The most prevalent drugs to treat MS comprise glucocorticoids, corticosteroids, intravenous immunoglobulins, interferons, and immunosuppressants such as natalizumab and azathioprine. However, their effects are limited, and long-term use is associated with serious side effects and complications that confer an additional burden on patients (Luna et al., 2020). Therefore, innovative drugs that could delay the onset, or halt the progression of MS are essential.

The pathological mechanisms and treatment of MS have been investigated using mature mouse models of experimental autoimmune encephalomyelitis (EAE) that simulates the key features of human MS (Bolton and Smith, 2018). Lymphocytes, macrophages and dendritic cells, are involved in the pathogenesis of MS, and are thought to form lesions *via* different and interacting mechanisms (Jolivel et al., 2013; Wang et al., 2019; Schafflick et al., 2020). Among these immunocytes, infiltrating microglia/macrophages are major effectors of inflammation and demyelination in MS and EAE (Hao et al., 2021; Lyu et al., 2021). In response to various stimuli, activated microglia/macrophages undergo significant morphological and functional changes and exhibit two phenotypes, i.e., inflammatory M1 and anti-inflammatory M2 that respectively release pro- and anti-inflammatory cytokines (Lyu et al., 2021). The upregulated M1-like phenotype secretes classic inflammatory mediators, such as tumor necrosis factor-alpha (TNF-α) and interleukin-12 (IL-12), and also inducible nitric oxide synthase (iNOS) during the early stages of disease development. These pro-inflammatory factors damage the integrity of myelin and promote its destruction. The M2-like phenotype notably upregulates anti-inflammatory mediators such as arginase-1 (Arg-1), interleukin-10 (IL-10), and CD206 (Leuti et al., 2021). Considering the different role of M1and M2 phenotype of microglia/macrophages in inflammatory response, regulating the transformation of M1 phenotype to M2 phenotype might be helpful for MS. Therefore, new drugs are needed that could promote the polarization of microglia/macrophages from pro-to the anti-inflammatory phenotype and thus be an effective strategy for treating EAE. Activated microglia and infiltrated macrophages are particularly enriched around white matter lesions in the central nervous system. These macrophages can produce a large amount of reactive oxygen (ROS) and nitrogen (RNS) species that damage the numerous axons and oligodendrocytes associated with the clinical course of MS (Pegoretti et al., 2020; Yin et al., 2022). Activated microglia/macrophages can activate the complement pathway, produce pro-inflammatory cytokines, release excitatory amino acids, and produce free radicals (Zhang J. et al., 2022).

Excessive ROS production exerts deleterious effects on lipids, proteins, and nucleic acids, leading to impaired cell function (Chen Y. et al., 2022). Under physiological conditions, ROS/RNS are cleared by antioxidant defense enzymes such as superoxide dismutase (SOD), catalase (CAT) and peroxidase and non-enzymatic compounds. Under pathological conditions, levels of ROS/RNS are elevated during inflammatory processes. These cause mitochondrial respiratory chain dysfunction and overstimulated glutamate NMDA receptors, which lead to low activities of oxidative scavengers such as cytosolic SOD and CAT, and significantly increased levels of lipid peroxide products, such as malondialdehyde (MDA) and 4-hydroxynonenal (4HNE) (Chen X. et al., 2022; Xue et al., 2022). Lipid peroxidation might be the main factor in free radical-mediated central nervous system damage. When excessive ROS cannot be neutralized by the antioxidant defense system, ROS metabolites can lead to protein and lipid peroxidation and DNA alkylation, which are also closely related to the occurrence of MS (Devanney et al., 2020)

The nuclear factor kappa B (NF-κB) is a family of transcription factors that regulate the expression many genes involved in cell death, inflammation, proliferation, and differentiation (Wang et al., 2022). Activation of NF-κB correlates with immune-associated genes that are upregulated in the serum, brain, and spinal cord of patients who have MS compared with healthy individuals (Soltanmoradi et al., 2021). Moreover, increased levels of NF-κB are associated with the course of MS (Jie et al., 2021). After organismal injury, excessive ROS accumulation results in binding to pattern recognition receptors, which might act as initiating signals to regulate NF-κB translocation to the nucleus and activate the NOD-like receptor family pyrin domain containing 3 (NLRP3) inflammasome, thus inducing IL-1β and IL-18 secretion, and inflammation (Peng et al., 2020). The NLRP3 inflammasomes are large protein complexes consisting of NLRP3, ASC, and caspase-1 (He et al., 2022). It is the most extensively studied type of inflammasome in the CNS and has been assessed in microglia (Chu et al., 2019). Moreover, NLRP3-associated pyroptosis protein is expressed in astrocytes, microglia, and neurons in EAE models (Hou et al., 2020; Zhang Q. et al., 2022; Song et al., 2022). Studies have shown that the activation NLRP3 inflammasomes induces the production of inflammatory cytokines and thus promotes the migration of immunocyte to the CNS in the EAE model of MS (Braga et al., 2019; Zhang Q. et al., 2022). These findings suggested that NLRP3 inflammasomes are associated with MS progress. Therefore, blocking the NLRP3 signaling pathway and pyroptosis can reduce neuroinflammation and thus alleviate disease progression.

We developed a novel recombinant globular protein flagellin A from *Legionella pneumophila* flagella (FLaAN/C). This biological agent is now an independent intellectual property owned by our team. It was designed by linking the C- and N-terminal ends of the flagellin to facilitate the reduction of flagellin’s side effects. In our previous studies, FLaAN/C regulates cell functions important for radiation protection by regulating the expression of multiple cytokines, and regulates the inflammatory response by inhibiting NF-κB signaling pathway. For example, FLaAN/C inhibits caspase-1-dependent pyroptosis by reducing NLRP3 inflammasome activity, and thus effectively mitigate radiation-induced intestinal injury (Wu et al., 2018). It significantly reduces radiation-induced pathology and improves the survival of mice after whole-body irradiation compared with controls. In addition, FLaAN/C upregulates miR-142a-3p, inhibits IRAK1/NF-κB signaling pathway in intestinal cells, thereby alleviating radiation-induced pyroptosis (Liu et al., 2022). Therefore, FLaAN/C inhibits inflammatory responses and oxidative stress. However, the role of FLaAN/C in the regulation of inflammasome-associated neuroimmunity remained unknown.

We aimed to determine the anti-demyelinating and anti-neuroinflammatory effects of FLaAN/C and its effects in an EAE mouse model. We also investigated the effects of FLaAN/C on microglial/macrophage polarization and NF-κB/NLRP3 signaling pathway. Our findings provided evidence for FLaAN/C as a new candidate therapeutic for MS.

## Materials and methods

### Establishment of the EAE model

**FIGURE 1:**
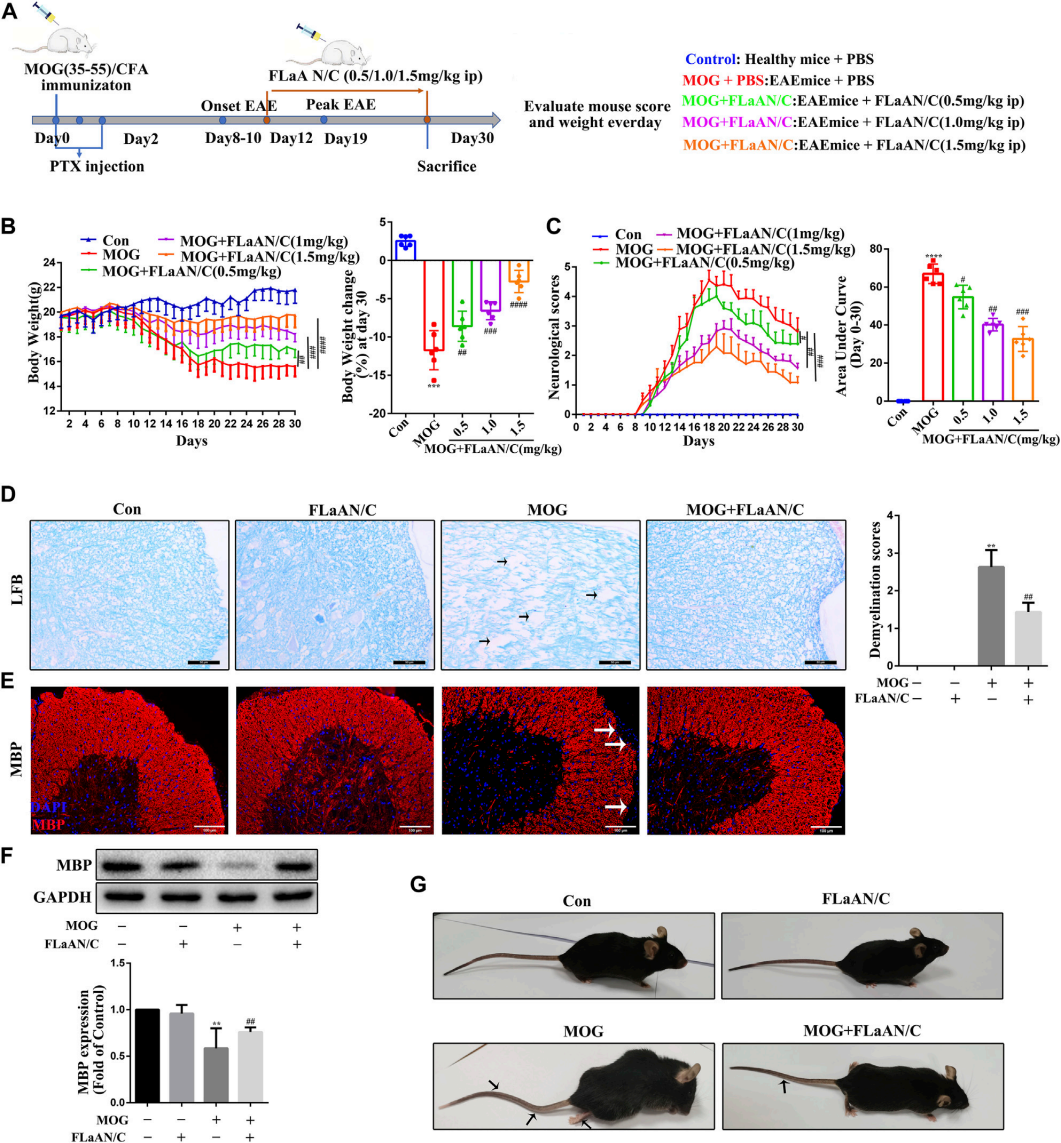
FLaAN/C reduces symptoms in EAE mice. **(A)** Study procedure. **(B)** Weight-versus-time curves of all experimental groups. **(C)** Clinical behavior scores for typical symptoms of EAE. **(D)** LFB staining showing demyelination in the spinal cord and the demyelination scores (The arrows present myelin injury and loss, scale bars:50 μm). **(E)** Immunofluorescence staining of MBP in the spinal cord (The arrows present myelin injury and loss, scale bars:100 μm). **(F)**Western bloting of MBP protein expression of the spinal cord. **(G)** Representative images show behavioral symptoms of EAE mice in each group (Black arrows present limp tail or hind limb paralysis). Paraffin sections of spinal cord tissue used for immunofluorescence staining. Data are shown as means ± SD (n = 6 per group). *p < 0.05, **p < 0.01, ***p < 0.001, ****p < 0.0001, ^#^ *p* < 0.05, ^##^ *p* < 0.01, ^###^ *p* < 0.001, ^####^ *p* < 0.0001. * vs. Control group; ^#^ vs. MOG group.

The Animal Policy and Welfare Committee of Chengdu Medical College approved all animal experiments (Approval ID: 2,021,711). Seven-week-old female C57/BL6 mice (weight, 18–21 g) (GemPharmatech Co., Ltd., Chengdu, China) and *Nlrp3* ^−/−^ mice on a C57BL/6 (Weishanglide Biotechnology Co., Ltd., Beijing, China) were raised at the Animal Experimental Center of Chengdu Medical College. We established the EAE model as described (Yu et al., 2020). Briefly, 100 μl of Complete Freund’s Adjuvant (CFA; Chondrex, Woodinville, WA, United States) was ultrasonically emulsified with 400 μg of Mycobacterium tuberculosis H37Ra (BD Biosciences, San Jose, CA, United States). Thereafter, 200 μg of myelin oligodendrocyte glycoprotein 35–55 (MOG35-55) peptide (Hooke Laboratories, Lawrence, MA, United States) mixed with 100 μl of PBS and the emulsion, was subcutaneously injected into the mice (immunization day 1). On that day and on post-immunization day 3, 300 ng of pertussis toxin (PTX; List Biological Laboratories Inc., Campbell, CA, United States) was injected i.p. (Figure 1A).

### FLaAN/C treatment

The preparation of the FLaAN/C recombinant protein was carried out by BGI Genomics (Shenzhen, China). Detailed preparation process for the FLaAN/C recombinant protein is provided in the supplementary material.The mice were randomly assigned to receive phosphate buffered saline (PBS; control n = 6), MOG + PBS (n = 6), or MOG + FLaAN/C (0.5, 1.0 and 1.5 mg/kg injected intraperitoneally (i.p.) daily from post-immunization day (p.i.d) 12 until p.i.d. 30; n = 6 each). On p.i.d. 30, all mice from each group were euthanized and spinal cord and blood were collected. The FLaAN/C treatment concentration was based on our pre-experiment: high-dose (10 mg/kg) single administration and low-dose (0.25 mg/kg) multiple administration. The pre-experiment results showed that the high dose (10 mg/kg) single administration exceeded the tolerance range of mice, and the mice died. Low-dose multiple administrations (0.25 mg/kg) had no significant therapeutic effect on EAE mice. In addition, clinical dosing regimens usually choose low-dose multiple administration. In this study, to better simulate clinical medication, we first determined the concentration gradient of FLaAN/C (0.5, 1.0, 1.5 mg/kg), and then screened the optimal treatment concentration of FLaAN/C (1.5 mg/kg). Therefore, we finally chose FLaAN/C (1.5 mg/kg) for subsequent experiments.

### Body weight and behavioral assessment

We weighed the mice daily and scored their clinical behavior from 0-5 according to the respective criteria for EAE: 0, no clinical symptoms; 1, the tail tension disappeared or the gait was awkward; 2, weakness in hind limbs; 3, paralysis of hind limbs; 4, paralysis of hind limbs and weakness in anterior limbs; 5, near-death state (Yu et al., 2020; Zilkha-Falb et al., 2020; Kim et al., 2021; Cai et al., 2022).

### Measurement of anti-oxidant activity

We measured SOD (S0101S), MDA (S0131S), and CAT (S0051) activities in mouse serum using the respective kits (Beyotime Biotechnology, Shanghai, China) as described by the manufacturer.

### Cytokine quantitation

Serum levels of the inflammatory cytokines TNF-α, IL-10, IL-8, IL-17, IFN-γ, IL-6, IL-1β, and IL-18 in mice were determined using enzyme-linked immunoassay (ELISA) kits (MIBIO Biotechnology, shanghai, China) as described by the manufacturer.

### Assays of caspase-1 activity

We evaluated caspase-1 activity in mouse spinal cord tissue using caspase-1 activity assay kit (C1102; Beyotime Biotechnology).

### Western bloting

Proteins were extracted from crushed spinal cord tissue by whole-cell lysis or using a nucleocytoplasmic protein extraction kit (KeyGEN BioTECH, Nanjing, China), and then centrifuged at 4°C (13,000 × *g*) for 15 min. Protein concentrations were determined in supernatants using BCA protein assay kits (Beyotime Biotechnology). The proteins (40 μg) were resolved by sodium dodecyl sulfate-polyacrylamide gel electrophoresis (SDS-PAGE), then transferred to PVDF membranes. Nonspecific antigen binding on the membranes was blocked by incubation 1 h at room temperature with 5% skimmed milk. Thereafter, the membranes were incubated overnight at 4°C with the following primary antibodies diluted 1:1000: GAPDH (10494-1-AP), TNF-α (17590-1-AP), CD86 (13395-1-AP), CD206 (18704-1-AP), Arginase-1 (16001-1-AP), IL-10 (D13A11), MBP (10458-1-AP), iNOS (22226-1-AP), nuclear factor of kappa light polypeptide gene enhancer in B-cells inhibitor, alpha IκBα (10268-1-AP), ASC 67494-1-Ig), IL-18 (10663-1-AP), lamin B1 (12987-1-AP), and NF-κB-p65 (10745-1-AP) all from Proteintech Group Inc., Rosemont, IL, United States), NLRP3 (ab270449), caspase-1 p20 (ab179515), IL-1β (ab216995), gasdermin D -N (GSDMD-N; ab219800), phosphorylated p-IκBα (ab133462), and NF-κB-p-p65 (ab194726) (all from Abcam, Cambridge, UK). The membranes were incubated in 1:8000-diluted horseradish peroxidase-conjugated secondary antibodies (SA00001-2; Proteintech) at 37°C for 1.5 h. Bands were visualized using a chemiluminescence horseradish peroxidase substrate (Merck Millipore, Burlington, MA, United States) and quantified using Quantity 5.2 (Bio-Rad Laboratories Inc., Hercules, CA, United States). Relative immunoreactivity was calculated according to gray values and normalized to that of the reference protein (GAPDH) using ImageJ (National Institutes of Health [NIH], Bethesda, MD, United States).

### Staining with hematoxylin and eosin (HE) and luxol fast blue (LFB)

We detected inflammatory cell infiltration of spinal cord tissue by staining with HE. Formalin-fixed, paraffin-embedded samples were stained with LFB using a kit (Solebo Biotech, Beijing, China). Six mice in each group were scored for inflammation and demyelination according to the following criteria. For inflammation: 0 = non-inflammatory cells; 1 = a small amount of scattered inflammatory cells; 2 = organization of inflammatory infiltrates around blood vessels; 3 = extensive perivascular cuffing with extension into parenchyma. For demyelination: 0 = none; 1 = rare foci; 2 = several demyelination areas; 3 = large areas of demyelination (Yu et al., 2020; Kim et al., 2021; Cai et al., 2022).

### Immunofluorescence staining

Paraffin blocks of spinal cord and brain tissue were cut into 5-µm thick sections, dewaxed with a graded ethanol series, and sealed with 5% fetal bovine serum for 30 min. The sections were incubated overnight at 4°C with the following primary antibodies (1:300): MBP (10458-1-AP), CD11b (ab1211), 4HNE (ab48506), 3NT (5411, Millipore), NLRP3 (ab270449), ASC (67494-1-Ig), and NF-κB (10745-1-AP). The sections were washed with PBS, then incubated with anti-Cy3 goat anti-rabbit IgG (H + L, A0516) or FITC goat anti-mouse IgG (H + L, A0568; Beyotime Biotechnology) secondary antibodies (1:200) at room temperature for 2 h. Then, the nuclei were stained with DAPI (1:10,000) for 8 min. Random images were photographed with a fluorescence microscope at ×40 magnification.

### Nuclear and cytoplasmic protein extraction

Nuclear and cytoplasmic proteins were extracted using a kit (P0027; Beyotime Biotechnology), then analyzed by western blotting.

### Co-immunoprecipitation

Lysed spinal cord tissue were shaken slowly with primary antibody overnight at 4°C, then complexes were immunoprecipitated using protein A + G agarose (P2055-10 ml; Beyotime Biotechnology) as described by the manufacturer.

### Quantitation of oxidative stress

Levels of ROS were detected by staining spinal cord and brain tissue with dihydroethidium (DHE) (S0063, Molecular Probe; Solarbio, Beijing, China). Paraffin slices were dewaxed and dehydrated in a graded ethanol series and washed with PBS (pH 7.4). Nonspecific antigen binding was blocked with 5% fetal bovine serum for 40 min, then stained with 5 mmol/L DHE in PBS for 30 min. Random images of brain or spinal cord sections were acquired using an XI 71 fluorescence microscope (Olympus, Tokyo, Japan) at ×20 magnification. The intensity of emitted fluorescence was analyzed using Image.

### Reverse transcription-quantitative polymerase chain reaction (RT-qPCR)

Total RNA extracted from mouse spinal cord tissue using a kit (Solarbio) was reverse transcribed into cDNA using iScript cDNA synthesis kit (BioRad Laboratories Inc., Hercules, CA, United States). The mRNA levels of *Il-8, TNF-*α*, Il-6, Il-10, Il-1β, Il-18, Il-17, IFN-γ, caspase-1,* and *GSDMD* were determined by qPCR using SYBR Green SuperMix (Bio-Rad Laboratories Inc). Expression relative to that of the internal reference β-actin (*Actb*) was calculated using the 2^−^^ΔΔCT^ method. Table 1 shows the synthesized primers (Shenggong, Shanghai, China) and the sequences are shown in Table 1.

**TABLE 1:** Primer sequence information.

Gene	Forward primer (5′F0223′)	Reverse primer (5′F0223′)
*Casp1*	CATCCTGTCAGGGGCTCACTTTTC	CTATCAGCAGTGGGCATCTGTAGC
*Gsdmd*	CGATGGGAACATTCAGGGCAGAG	ACACATTCATGGAGGCACTGGAAC
*Il1β*	CAAGAGCTTCAGGCAGGCAGTATC	AGGTCCACGGGAAAGACACAGG
*Il18*	GGCTGCCATGTCAGAAGACTCTTG	AGTGAAGTCGGCCAAAGTTGTCTG
*Il10*	CACTGCTATGCTGCCTGCTCTTAC	TGGGAAGTGGGTGCAGTTATTGTC
*Il8*	CATGGGTGAAGGCTACTGTTGGC	GCTTCATTGCCGGTGGAAATTCC
*Tnfα*	TCTACTGAACTTCGGGGTGATCGG	GTGGTTTGTGAGTGTGAGGGTCTG
*Ifnγ*	AGGAACTGGCAAAAGGATGGTGAC	GTTGTTGCTGATGGCCTGATTGTC
*Il17*	GCCAAGGACTTCCTCCAGAATGTG	TGGAACGGTTGAGGTAGTCTGAGG
*Il6*	CTTGGGACTGATGCTGGTGACAAC	AGGTCTGTTGGGAGTGGTATCCTC

### Statistical analysis

All experiments were repeated independently three times. All data were statistically analyzed using Prism7.0 (GraphPad Software Inc., San Diego, CA, United States). Pairs of groups were compared using two-tailed Student t-tests, and three or more groups were compared using a one-way ANOVA. Data are expressed as means ± SD, and values with *p* < 0.05 were considered statistically significant.

## Results

### FLaAN/C delayed EAE peak and relieved symptoms

To assess the therapeutic effects of FLaAN/C on EAE severity, mice were injected daily with PBS or with FLaAN/C at doses of 0.5,1.0, or 1.5 mg/kg/day/i.p, from day 12 post first immunization until the end of the study at day 30 (Figure 1A). Daily weight changes and clinical behavioral scores were recorded. More body weight was lost by the MOG-treated, than control mice, but FLaAN/C dose-dependently improved this. The results showed that FLaAN/C treatment significantly delayed the detrimental effects of EAE on body weight (Figure 1B). Furthermore, clinical scores were assessed as the area under the curve (AUC). The clinical scores were higher in the MOG-treated mice than the control mice and FLaAN/C (0.5, 1.0, and 1.5 mg/kg/day) significantly reduced clinical score in a dose-dependent manner (Figure 1C). These data indicated that the best concentration for FLaAN/C was 1.5 mg/kg and we selected this concentration in subsequent experiments. Spinal cord demyelination was more severe in mice with MOG, than that in mice given PBS (Figure 1D), and FLaAN/C (1.5 mg/kg) treatment significantly attenuated this phenomenon. Similarly, IF staining and western bloting showed that FLaAN/C significantly increased MBP expression in MOG mice (Figure 1 E and F). At the same time, we also found that MOG mice showed severe clinical symptoms with loose tails and completely paralyzed hind limbs, while EAE mice treated with FLaAN/C showed only weak and drooping tails (Figure 1G). These results indicated that FLaAN/C prevented myelin loss associated with EAE progression.

### FLaAN/C shifted the M1 to the M2 phenotype in spinal cord of mice with EAE

**FIGURE 2:**
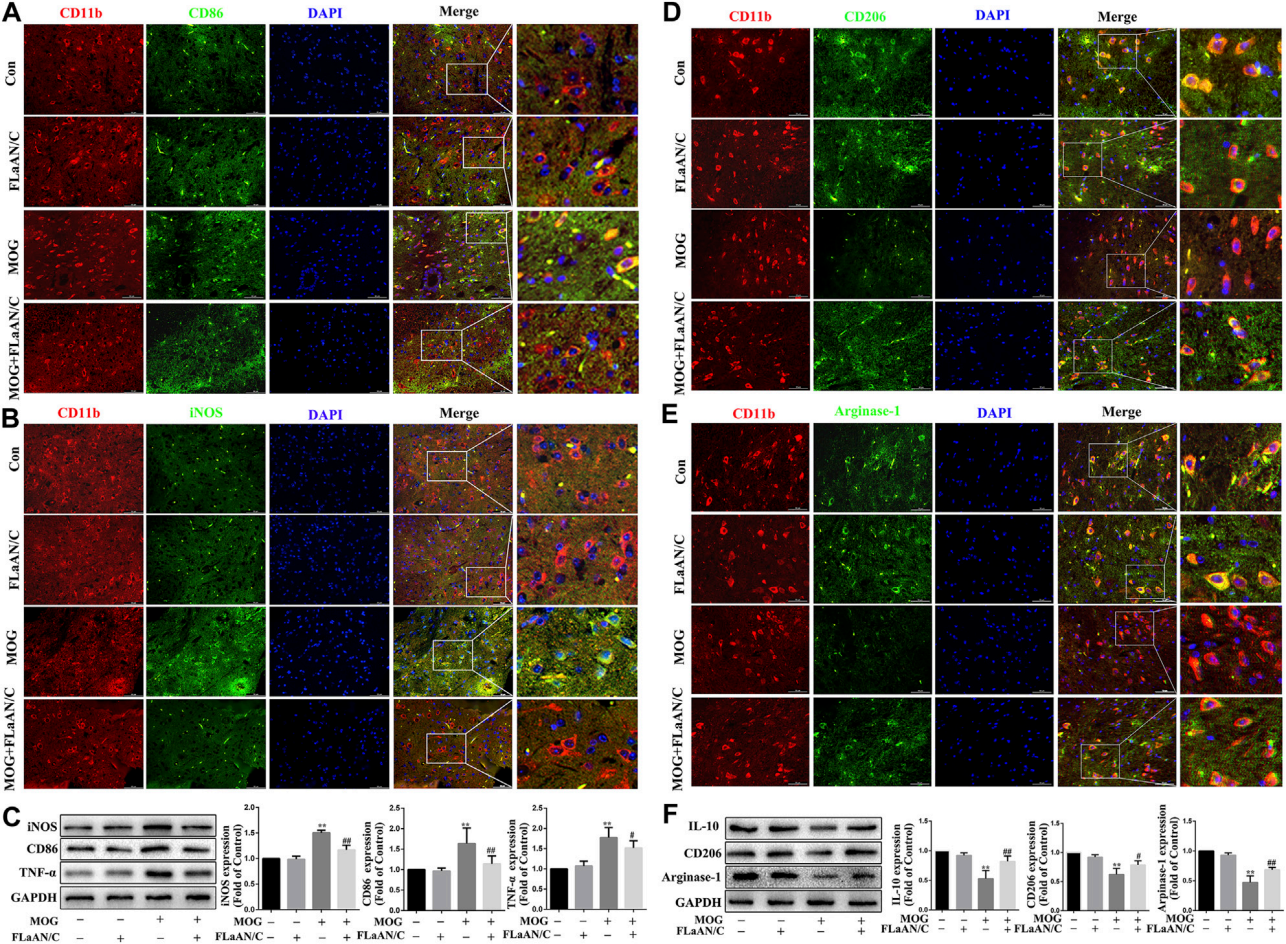
FLaAN/C shifted M1 to M2 phenotype in spinal cord of mice with EAE. The mice were euthanized on post-immunization day 30. The spinal cord tissue of each group of mice were isolated and embedded in paraffin.Immunofluorescence was used to detect the colocalization of CD11b with M1 markers (iNOS and CD86) and M2 markers (Arginase-1 and CD206) in spinal cord tissue.The representative images show the expression levels of CD86^+^ **(A)**, iNOS^+^ **(B)**, CD206+**(D)** and Arginase-1^+^ **(E)** in CD11b^+^ cells (scale bars: 50 μm). **(C)** The protein expression levels of iNOS, CD86 and TNF-α in the spinal cord of each group was detected by western bloting. **(F)** The protein expression levels of Arginase-1, CD206 and IL-10 in the spinal cord of each group were detected by western bloting. Data are shown as means ± SD (n = 6 per group). *p < 0.05, **p < 0.01, ***p < 0.001; ^#^ *p* < 0.05, ^##^ *p* < 0.01, ^###^ *p* < 0.001. * vs. Control group; ^#^ vs. MOG group.

The polarization of microglia/macrophages play key roles in the response to inflammation. To evaluate the effect of FLaAN/C on the polarization of microglia/macrophages in spinal cord of mice with EAE, we used immunofluorescence to detect the colocalization of CD11b with M1 markers (iNOS and CD86) and M2 markers (Arginase-1 and CD206) in spinal cord tissue to analyze the microglia/macrophages phenotype. The results showed that the coexpression of CD86 and CD11b was dramatically lower in FLaAN/C-treated mice with EAE than in the PBS-treated EAE mice (Figure 2A), and FLaAN/C treatment markedly decreased the foci of iNOS^+^CD11b^+^ in the EAE mouse spinal cord compared to those of PBS-treated mice with EAE (Figure 2B). Consistent with the immunofluorescence staining results, the western bloting datas showed that the protein levels of M1 phenotypic markers (iNOS, TNF-α, and CD86) were significantly increased in the MOG group compared with the control group, whereas the protein expression levels of iNOS, TNF-α, and CD86 were significantly decreased in the MOG + FLaAN/C group (Figure 2C). In addition, the foci of CD206^+^CD11b^+^ and Arginase-1^+^CD11b^+^ were significantly increased in spinal cord of FLaAN/C-treated MOG mice compared with the MOG mice treated with PBS (Figures 2D,E). Besides, protein levels of the M2 phenotype markers (IL-10, Arginase-1, and CD206) were substantially increased in the FLaAN/C-treated MOG mice compared with PBS-treated MOG mice (Figure 2F). These results indicated that the anti-inflammatory effect of FLaAN/C in the spinal cord may be related to its regulation of microglia/macrophage phenotype.

### FLaAN/C suppressed immune cell infiltration and proinflammatory cytokine production in spinal cord of EAE mice

**FIGURE 3:**
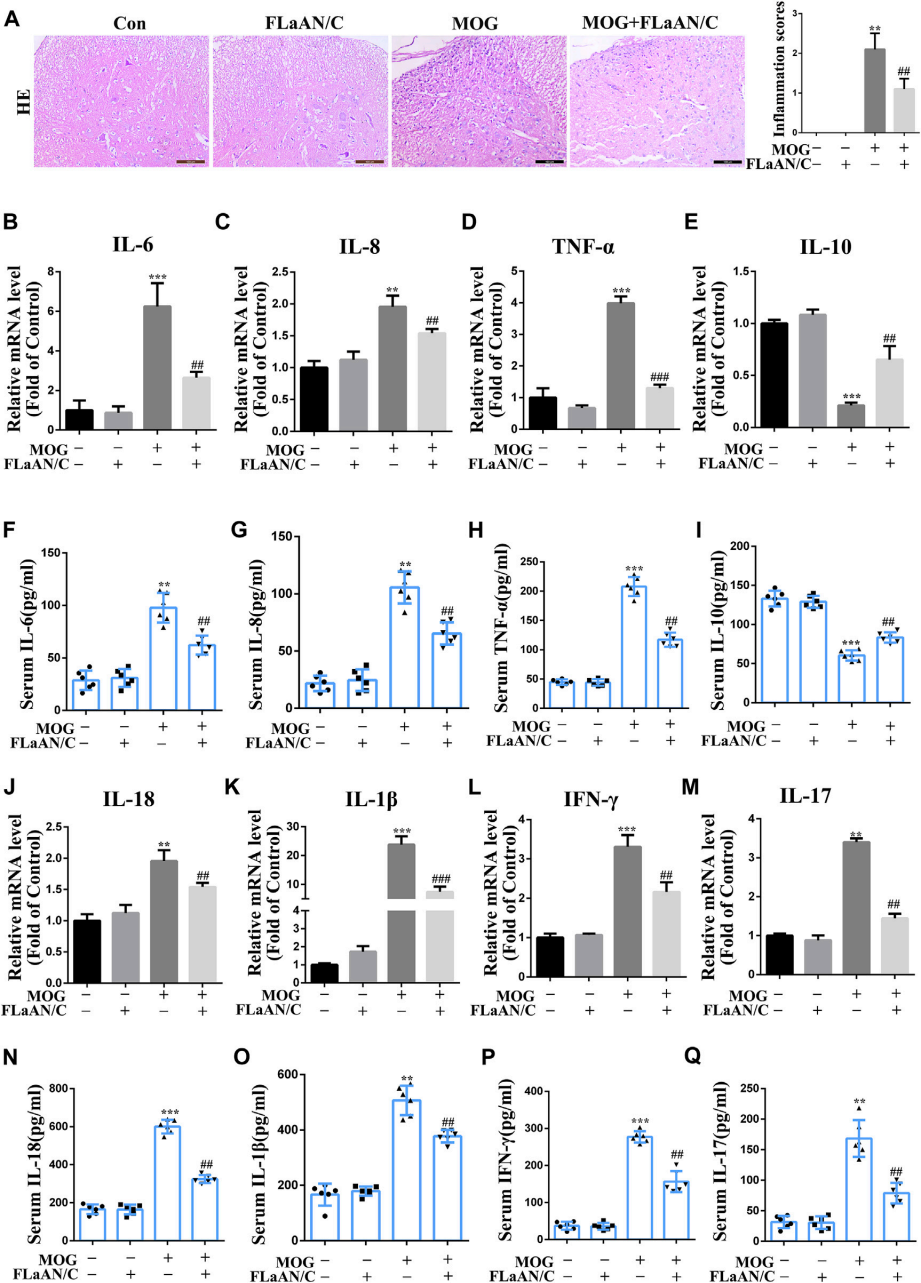
Effects of FLaAN/C on inflammation in mice with EAE. **(A)** H&E staining was performed on paraffin-embedded mouse spinal cord sections (scale bars: 100 μm). **(B–E)** The mRNA expression of IL-6 **(B)**, IL-8 **(C)**, TNF-α **(D)**, and IL-10 **(E)** were quantitated by qRT-PCR. **(F–I)** Serum levels of IL-6 **(F)**, IL-8 **(G)**, TNF-α **(H)**, IL-10 **(I)** were measured by ELISA. (J–M) The mRNA expression of IL-18 **(J)**, IL-1β **(K)**, IFN-γ **(L)**, and IL-17 **(M)** were quantitated by qRT-PCR. **(N–Q)** Serum levels of IL-18 **(N)**, IL-1β **(O)**, IFN-γ **(P)**, and IL-17 **(Q)** were measured by ELISA. Data are shown as means ± SD (n = 6 per group). *p < 0.05, **p < 0.01, ***p < 0.001; ^#^ *p* < 0.05, ^##^ *p* < 0.01, ^###^ *p* < 0.001. * vs. Control group; ^#^ vs. MOG group.

Considering the important role of inflammatory cytokines in the development of EAE, we evaluated the anti-inflammatory effect of FLaAN/C. Our results showed that MOG significantly increased the number of inflammatory cells and inflammation scores in the spinal cord, however, FLaAN/C noticeably reduced both of these effects (Figure 1A). We also evaluated the levels of several cytokines by qRT-PCR in the spinal cord and found that the mRNA levels of pro-inflammatory cytokines IL-6, IL-8 and TNF-α were markedly increased in the spinal cord of MOG mice, while that of the anti-inflammatory cytokine IL-10 was decreased. Meanwhile, FLaAN/C reduced the mRNA levels of IL-6, IL-8 and TNF-α, and increased that of IL-10 (Figures 3B–E). In addition, we quantified the IL-6, IL-8, TNF-α and IL-10 levels in serum by ELISA assay and found that IL-6, IL-8 and TNF-α levels in serum were increased in MOG mice, and FLaAN/C reduced their upregulation. However, the expression level of IL-10 showed the opposite trend (Figures 3F–I). Furthermore, we quantified the IL-18, IL-1β, IFN-γ and IL-17 levels by qRT-PCR and ELISA assay, and found that MOG significantly upregulated the levels of IL-18, IL-1β, IFN-γ and IL-17, while FLaAN/C remarkably reduced the expression of them (Figure 3J-Q). These results convincingly demonstrated the anti-inflammatory effect of FLaAN/C.

### FLaAN/C significantly inhibited oxidative stress in EAE mice

**FIGURE 4:**
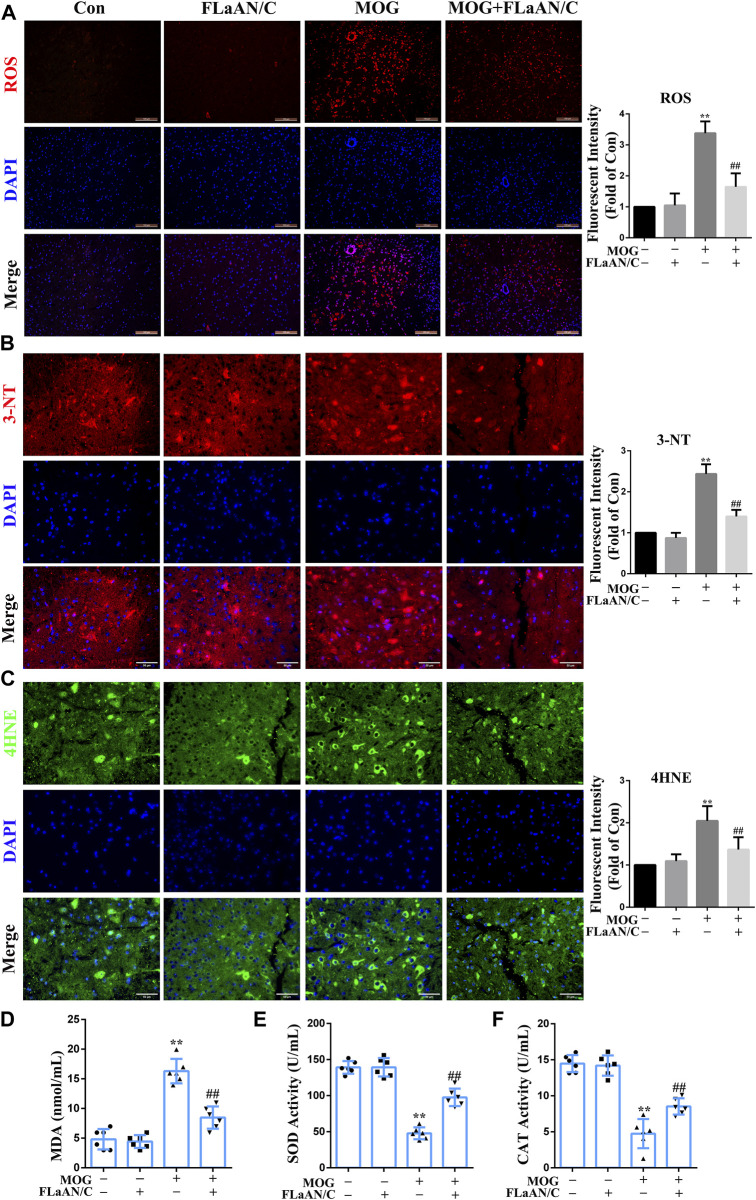
FLaAN/C alleviated MOG-induced oxidative stress in the spinal cord of EAE mice. **(A)** Abundance of reactive oxygen species (ROS) in spinal cord detected by DHE staining (scale bars: 100 μm). **(B–C)** Representative immunofluorescent images of 3-NT **(B)** and 4HNE **(C)** in the spinal cord of all experimental mice (scale bars:50 μm). **(D–F)** The activity levels of MDA **(D)**, SOD **(E)** and CAT **(F)** in the serum of each group of mice. Paraffin sections of spinal cord tissue used for immunofluorescence staining. Data are shown as means ± SD (n = 6 per group). *p < 0.05, **p < 0.01, ***p < 0.001; ^#^ *p* < 0.05, ^##^ *p* < 0.01, ^###^ *p* < 0.001. * vs. Control group; ^#^ vs. MOG group.

To clarify whether FLaAN/C exerts its anti-inflammatory effect through an antioxidant effect, we used DHE staining to evaluate the levels of ROS in the spinal cord. The results showed that ROS levels were significantly increased in MOG mice compared with the mice in control group, while FLaAN/C effectively inhibited ROS production (Figure 4A). Expression levels of superoxide dismutase (SOD) and catalase (CAT) antioxidant enzyme system mainly reflect the ability to physiologically remove oxygen free radicals. In addition, 3-nitrotyrosine(3-NT), 4-hydroxynonenal (4HNE), and malondialdehyde (MDA) are metabolites of oxidative stress and their expression levels mainly reflect the state of oxidative stress. In this study, we found that the levels of 3-NT, 4HNE, and MDA in MOG mice were reduced by FLaAN/C treatment (Figures 4B–D). Additionally, FLaAN/C can effectively increase the expression of SOD and CAT in MOG mice (Figures 4E,F).

### FLaAN/C inhibited NF-κB activation and prevented IκBα degradation and phosphorylation in EAE mice

**FIGURE 5:**
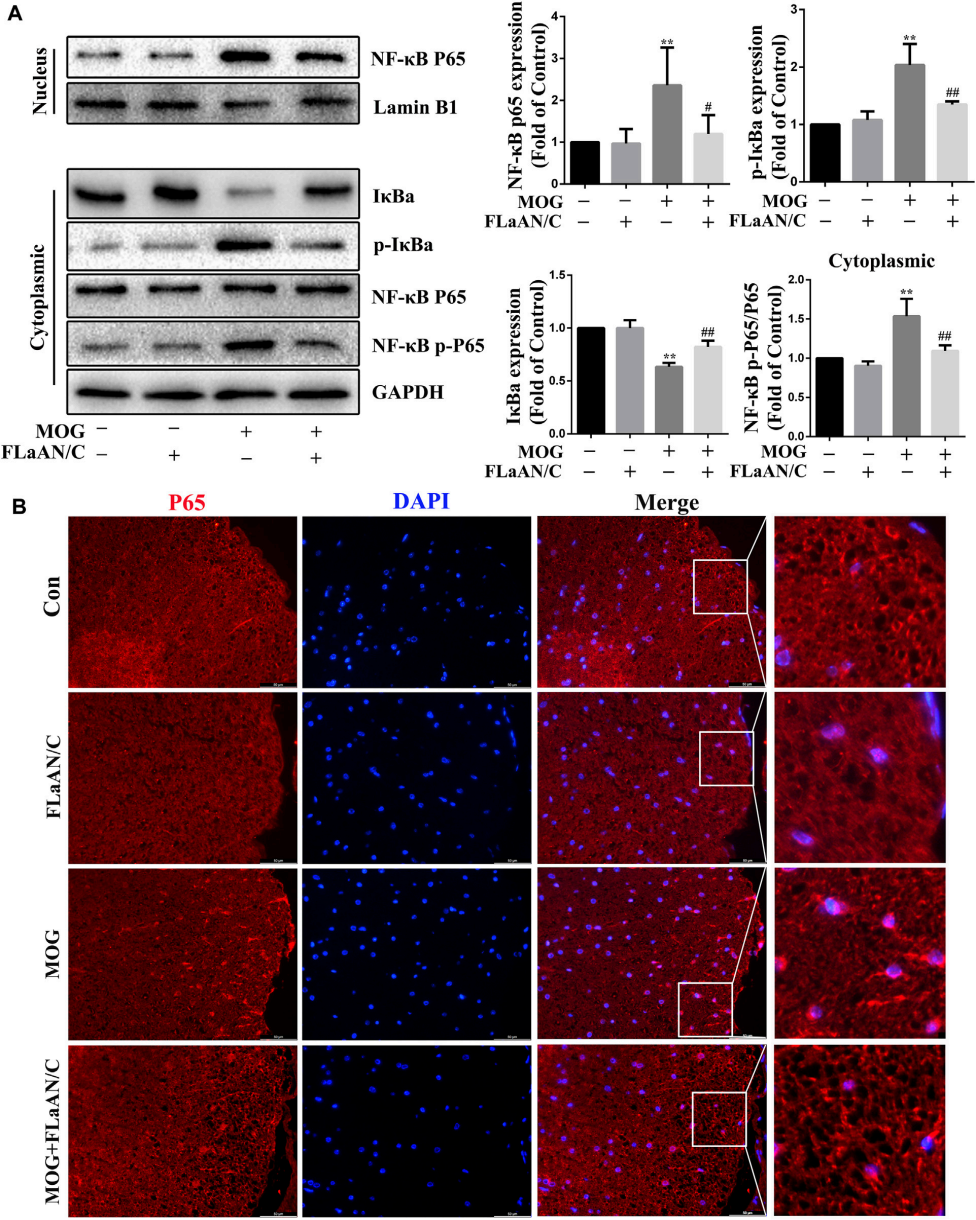
FLaAN/C inhibited NF-κB activation in mice with EAE. **(A)** Proteins levels of p-IκBα, IκBα, NF-κB-p65, and NF-κB-p-p65 in mouse spinal cord. **(B)** Representative immunofluorescence images of p65 in paraffin section of spinal cord tissue (scale bars:50 μm). Data are shown as means ± SD (n = 6 per group). *p < 0.05, **p < 0.01, ***p < 0.001; ^#^ *p* < 0.05, ^##^ *p* < 0.01, ^###^ *p* < 0.001. * vs. Control group; ^#^ vs. MOG group.

The transcription factor NF-κB is associated with the development of several neuroinflammatory diseases and its activation is the initiation signal for NLRP3 inflammasome (Cai et al., 2022). Thus, we detected the expression levels of NF-κB-related proteins in spinal cord tissue. Western bloting showed that the expression of p-IκBα, p-NF-κB, and NF-κB were significantly upregulated in the spinal cord of MOG mice, while IκBα expression was downregulated. Furthermore, FLaAN/C reversed these effects. Similarly, in the FLaAN/C-treated MOG group, the expression level of IκBα was significantly improved, while the expression of p-IκBα was significantly reduced (Figure 5A). Immunofluorescence staining revealed that the number of cells with activated p65 also increased in the MOG group. However, this phenomenon was reversed by FLaAN/C treatment (Figure 5B). These results indicates that FLaAN/C suppresses the NF-κB pathway.

### NLRP3-mediated pyroptosis promoted EAE development and progression

**FIGURE 6:**
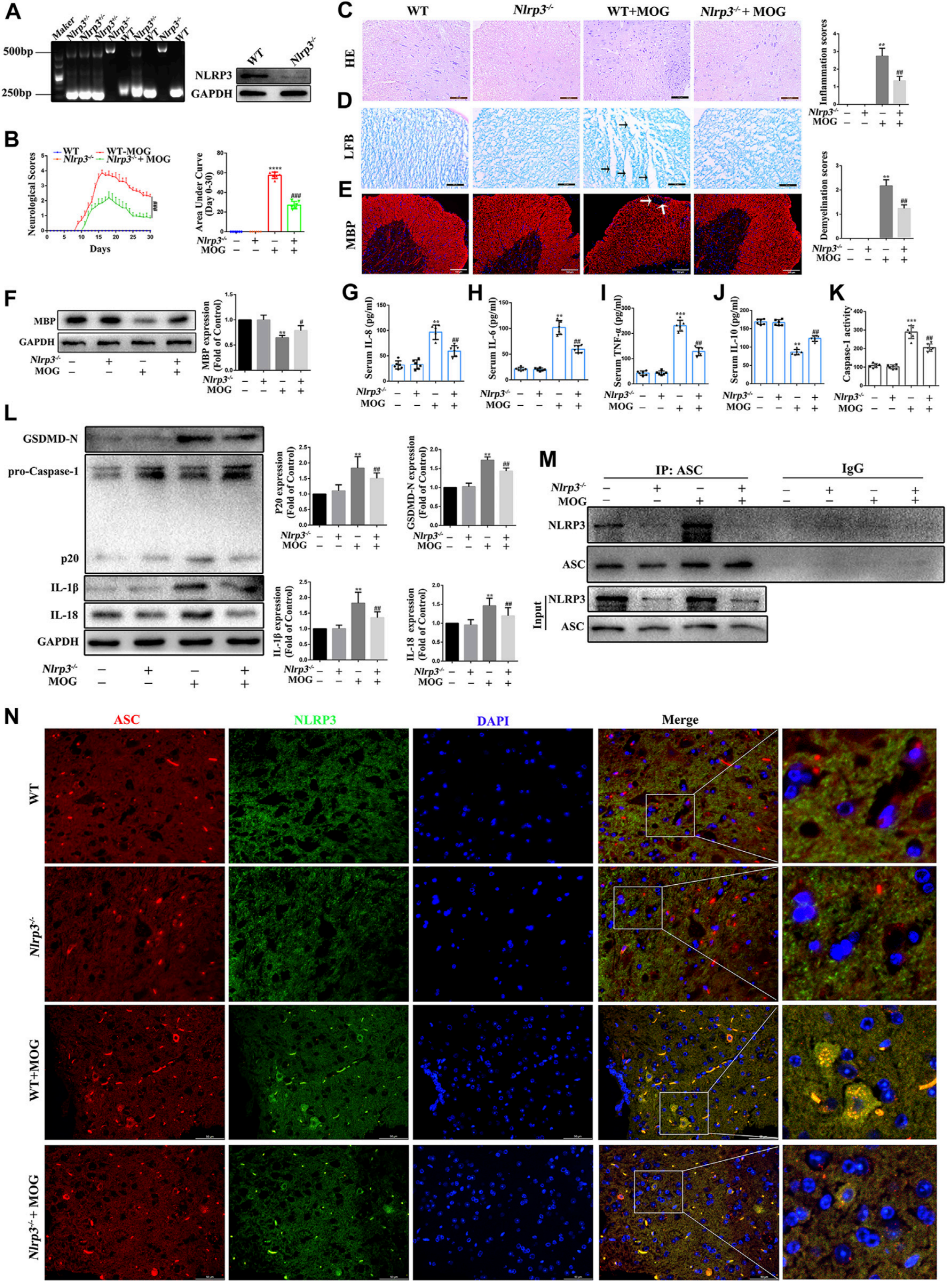
NLRP3 inflammasome mediated pyroptosis plays an important role in the pathogenesis of EAE **(A)** Identification of *Nlrp3* knockout (KO) mice. **(B)** Clinical behavioral scores and the area under the curve (AUC). **(C)** H&E staining was performed on paraffin-embedded mouse spinal cord sections (scale bars: 100 μm). **(D)** LFB staining was performed on paraffin-embedded mouse spinal cord sections (The arrows present myelin injury and loss, scale bars:50 μm). **(E)** Immunofluorescence staining of MBP in the spinal cord (The arrows present myelin injury and loss, scale bars:100 μm). **(F)** Evaluation of MBP protein expression in spinal cord by western blotting. **(G–J)** Serum inflammatory factor levels of IL-8 **(G)**, IL-6 **(H)**, TNF-α **(I)**, and IL-10 **(J)** are detected using ELISA. **(K)** Caspase-1 activity in mouse spinal cord was detected using the kit. **(L)** The protein levels of pyroptosis markers assessed using western blotting in spinal cord tissue. **(M)** Detection of NLRP3 and ASC complex formation in spinal cord by co-immunoprecipitation. **(N)** Representative image of co-localized NLRP3 and ASC with immunofluorescence staining in spinal cord tissue (scale bars: 50 μm). Paraffin sections of spinal cord tissue used for immunofluorescence staining. Data are shown as means ± SD (n = 6 per group). “-” for “Nlrp3^−/−^” represents wild type mice and “+” for “Nlrp3^−/−^” represents Nlrp3 knockout mice. *p < 0.05, **p < 0.01, ***p < 0.001; ^#^ *p* < 0.05, ^##^ *p* < 0.01, ^###^ *p* < 0.001.* vs. WT group; ^#^ vs. WT + MOG group.

The NLRP3 inflammasome plays a key role in innate immunity responses and inflammatory events, and is involved in the occurrence and progression of MS. In this study, we evaluated the role of NLRP3-mediated pyroptosis in the progression of EAE. First, we genetically identified *Nlrp3*-KO mice and screened for *Nlrp3* ^−/−^ homozygotes for subsequent experiments (Figure 6A). Then, we found that *Nlrp3* ^−/−^-MOG mice had significantly fewer signs of disease and correspondingly lower clinical scores than WT-MOG mice (Figure 6B). At the same time, the results of HE staining showed that MOG significantly increased the number of inflammatory cells in the spinal cord and the inflammation score, while *Nlrp3* ^−/−^-MOG mice were significantly reduced both effects (Figure 6C). Furthermore, LFB staining revealed a lower level of spinal cord demyelination in *Nlrp3* ^−/−^-MOG mice compared with WT-MOG mice (Figure 6D). Similarly, IF staining and western bloting showed that the protein levels of MBP were significantly lower in WT-MOG mice than in *Nlrp3* ^−/−^-MOG mice (Figures 6E,F). Furthermore, we detected the levels of inflammatory cytokines in the serum of mice by ELISA assay and found that the levels of pro-inflammatory cytokines TNF-α, IL-6, and IL-8 were significantly increased in WT-MOG mice, while the level of anti-inflammatory cytokine IL-10 was decreased (Figures 6G–J). Next, we detected the activity of caspase-1. The activity of caspase-1 in *Nlrp3* ^−/−^-MOG mice was lower than that in WT-MOG mice (Figure 6 K). Western bloting results showed that the protein levels of caspase-1 p20, GSDMD-N, IL-18 and IL-1β were increased in WT-MOG mice, but the expression levels of these proteins were significantly decreased in *Nlrp3* ^−/−^-MOG mice (Figure 6L). Additionally, co-immunoprecipitation analysis revealed more abundant NLRP3-ASC complexes in the spinal cord of WT-MOG mice (Figure 6M). This finding was confirmed by immunofluorescence staining, which showed that more cells were positive for both NLRP3 and ASC in the WT-MOG group (Figure 6N). Overall, these results showed that NLRP3-mediated pyroptosis plays an important role in the occurrence and development of EAE.

### FLaAN/C attenuated NLRP3-mediated pyroptosis in EAE mice

**FIGURE 7:**
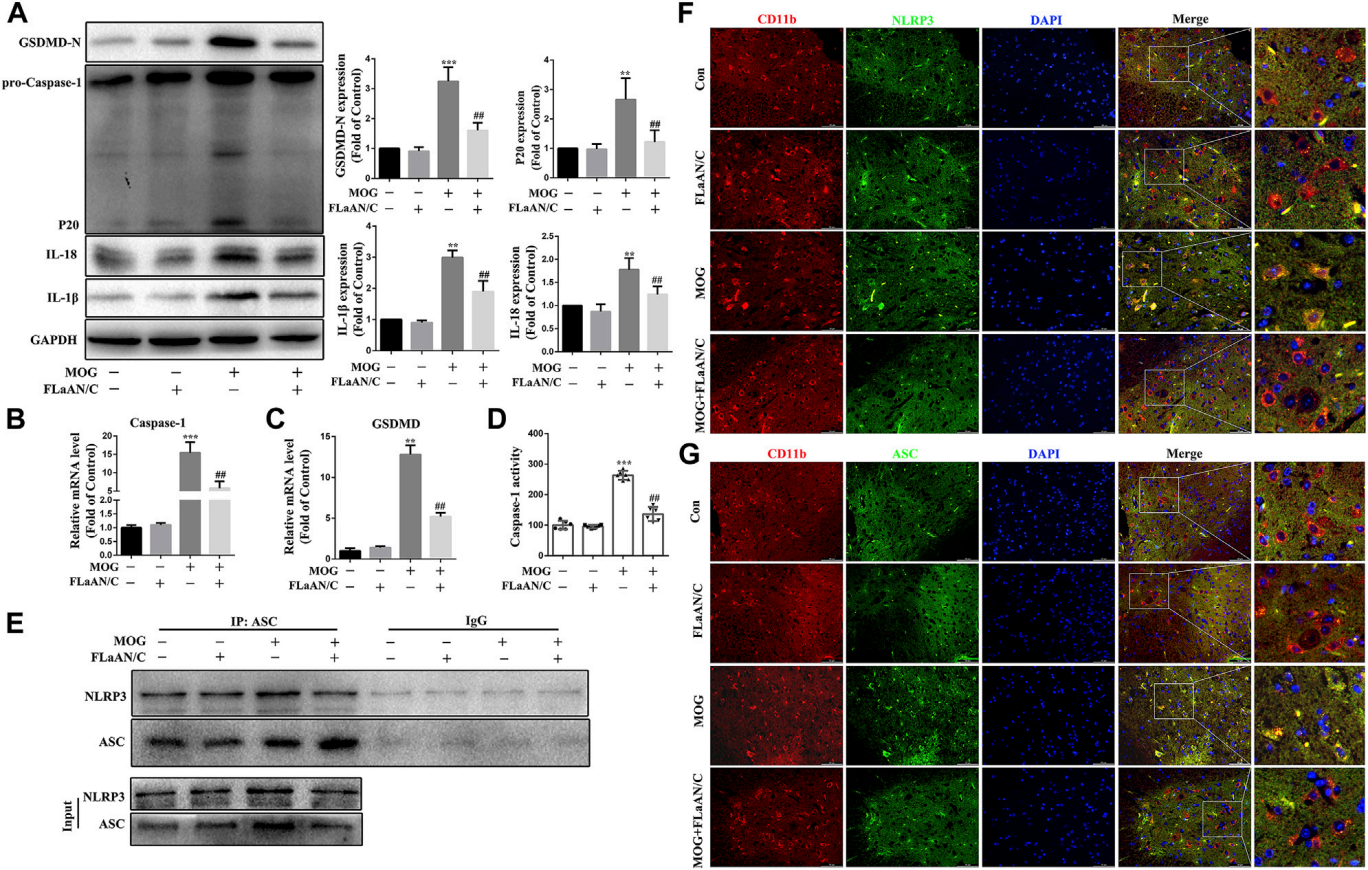
FLaAN/C attenuated NLRP3-mediated pyroptosis in EAE mice. **(A)** Abundance of caspase-1 p20, GSDMD-N, and IL-18 and IL-1β proteins in mouse spinal cord. **(B–C)** The mRNA expression of Caspase-1 **(B)** and GSDMD **(C)** were quantitated by qRT-PCR. **(D)** Caspase-1 activity in mouse spinal cord was detected using the kit. **(E)** Detection of NLRP3 and ASC complex formation in spinal cord by co-immunoprecipitation. **(F–G)** Representative images of NLRP3^+^ **(F)** and ASC^+^ **(G)** in CD11b^+^ cells in sections of paraffin-embedded spinal cord tissue (scale bars: 50 μm). Data are shown as means ± SD (n = 6 per group). *p < 0.05, **p < 0.01, ***p < 0.001; ^#^ *p* < 0.05, ^##^ *p* < 0.01, ^###^ *p* < 0.001. * vs. Control group; ^#^ vs. MOG group.

Considering the important role of NLRP3-mediated pyroptosis in EAE, we further explored whether FLaAN/C suppresses inflammatory responses in EAE mice by inhibiting NLRP3-mediated pyroptosis. We evaluated the protein levels of the NLRP3 inflammasome complex in the spinal cord by western blotting. And the results showed that the protein expression levels of caspase-1 p20, GSDMD-N, IL-18 and IL-1β in FLaAN/C-treated MOG mice were significantly lower than those in MOG mice (Figure 7A). The same trend was observed for the mRNA levels of caspase-1 and GSDMD detected by RT-qPCR (Figures 7B,C). Furthermore, FLaAN/C significantly decreased the activity of Caspase-1 in the spinal cord of MOG mice (Figure 7D). Next, co-immunoprecipitation assays revealed that the NLRP3-ASC complex was decreased in FLaAN/C-treated MOG mice (Figure 7E). Since NLRP3 and ASC are closely associated with the activation of NLRP3 signaling pathway, we further detected the expression levels of ASC and NLRP3 in the spinal cord. Immunofluorescence staining confirmed that FLaAN/C markedly decreased the foci of CD11b+NLRP3^+^ and CD11b^+^ASC^+^ in the MOG mice (Figures 7F,G). Altogether, these findings showed that FLaAN/C inhibited NLRP3 mediated pyroptosis.

## Discussion

Multiple sclerosis is a prevalent autoimmune inflammatory demyelinating disease of the CNS that significantly affects the quality of life of patients (Zilkha-Falb et al., 2020). Although there are many drugs available to treat MS, they are not entirely effective. Therefore, the pathogenesis of MS needs to be understood from several perspectives to generate more useful and comprehensive information that will help to improve and optimize of treatment options. Cascade neuroinflammation is a cause of MS, and once the CNS inflammatory response spreads, it can lead to severe and permanent neuronal damage (Zilkha-Falb et al., 2020). Therefore, inflammation control is the main goal of early disease treatment.

Extensive activation of microglia/macrophages is a crucial factor in mediating neuroinflammatory damage to the CNS. Microglia/macrophages comprise M1 and M2 phenotypes. Surface markers of microglia/macrophages recognized by antibodies that assess their activation, comprise CD11b, ionized calcium-binding adaptor molecule 1 (IBA1), CD68, and glycoprotein F4/80. M1 macrophages/microglia secrete the proinflammatory cytokines, IL-1β, IL-6, TNF-α, as well as CD16/32 (Fc gamma III and II receptors, respectively), and inducible nitric oxide synthase (iNOS), whereas the M2 phenotype is generally characterized by upregulated Arginase-1, IL-10, CD206, and CD163 (Cutolo et al., 2019). The phenotypes of microglia/macrophages include M1 phenotype and M2 phenotype. M1 and M2 can play different roles in MS through their secreted cytokines (Jäckle et al., 2020). Accordingly, regulating the polarization of microglia/macrophages from the pro-to the anti-inflammatory phenotype might help to treat inflammatory diseases of the CNS. For example, interleukin one receptor associated kinase M (IRAK-M) significantly improves EAE onset by downregulating the TLR4-MyD88 signaling pathway, which finally leads to differentiation to the M2 phenotype in microglia (Liu et al., 2019; Jäckle et al., 2020). Homeobox protein MSX-3 (MSX3) drives microglia polarization to the M2 phenotype and promotes oligodendrocyte precursor maturation and remyelination, thus preventing experimental EAE progression (Yu et al., 2015). We found that FLaAN/C promoted the shift of microglia/macrophages to an anti-inflammatory phenotype, as indicated by a lower abundance of the M1 marker CD86 and a higher abundance of the M2 marker CD206, accompanied by decreased iNOS and TNF-α secretion, and enhanced IL-10 and Arginase-1 release. Our results indicated that FLaAN/C treatment promoted the polarization of microglia/macrophages from M1 to M2 phenotype, thereby attenuating the inflammatory response in the spinal cord of EAE mice.

The CNS is highly vulnerable to oxidative stress due to high energy demand, mitochondrial activity, and polyunsaturated fatty acids. Hence, these features increase CNS susceptibility to typical neurodegenerative hallmarks linked with oxidative stress, which might cause CNS dysfunction in patients with MS (Steudler et al., 2022). Novel fatty acid-binding protein five and seven inhibitors ameliorate glial cell injury in EAE by decreasing oxidative stress levels (Cheng et al., 2021). Consistent with these findings, the present study showed that FLaAN/C might prevent ROS accumulation by downregulating 3NT, 4HNE, and MDA levels in EAE mice, while increasing SOD and CAT expression levels. Reactive oxygen species not only mediate oxidative stress but also promote the NF-κB signaling pathway which is widely involved in the occurrence of MS (Gutiérrez-Miranda et al., 2020; Lei et al., 2020). Furthermore, paricalcitol inhibits neuroinflammation and moderates MOG35-55-induced EAE progression by modulating NF-κB signaling both *in vitro* and *in vivo* (Zhang et al., 2020). Current approaches to treating MS, such as IFN-β and glatiramer acetate, significantly inhibit NF-κB activity (Zhao et al., 2018). Our results have consistently showed that FLaAN/C decreases the nuclear expression of NF-κB subunit p65 and the numbers of cells expressing activated NF-κB in mice with EAE.

Pyroptosis is an inflammatory cell death process that is activated by the NLRP3 inflammasome *via* a classical pathway. Emerging evidence suggests that NLRP3-mediated pyroptosis is functioning in the CNS of patients with MS and in mice with EAE (Khan et al., 2018). Moreover, the NLRP3 inflammasome is regulated by upstream components such as NF-κB and ROS (Yin et al., 2020). Pattern recognition receptors binding to their corresponding ligands, such as ROS, are the first signal to mediate translocation to the nucleus, thus initiating downstream NLRP3, Pro-IL-1β, and Pro-IL-18 transcription, and upregulating the expression of relevant inflammatory factors (Zhang et al., 2018; Li et al., 2021). The NF-κB/NLRP3 signaling pathway plays important roles in inflammatory diseases of the nervous system. For instance, Baicalin exerts neuroprotective effects by inhibiting activation of the GSK3β/NF-κB/NLRP3 signaling pathway in a rat model of depression (Zhang et al., 2018). We found that the NF-κB/NLRP3 signaling pathway is also involved in the occurrence of EAE. Demyelination and inflammatory cell infiltration were alleviated in *Nlrp3* ^−/−^-MOG mice. Moreover, *Nlrp3* ^−/−^-MOG mice have decreased activation and protein levels of caspase-1 p20 and GSDMD-N, as well as significantly reduced pyroptosis-related indicators, such as caspase-1 activation, and IL-18 and IL-1β levels. Similarly, FLaAN/C significantly decreased caspase-1 activation and inhibited the protein expression of IL-18, IL-1β, pro-caspase-1 p20 and GSDMD-N. Furthermore, FLaAN/C significantly inhibited the colocalization of NLRP3 and ASC and inhibited the activation of NLRP3 inflammasome. These results suggested that the therapeutic effects of FLaAN/C are exerted by inhibiting NLRP3 inflammasome-mediated pyroptosis.

**FIGURE 8:**
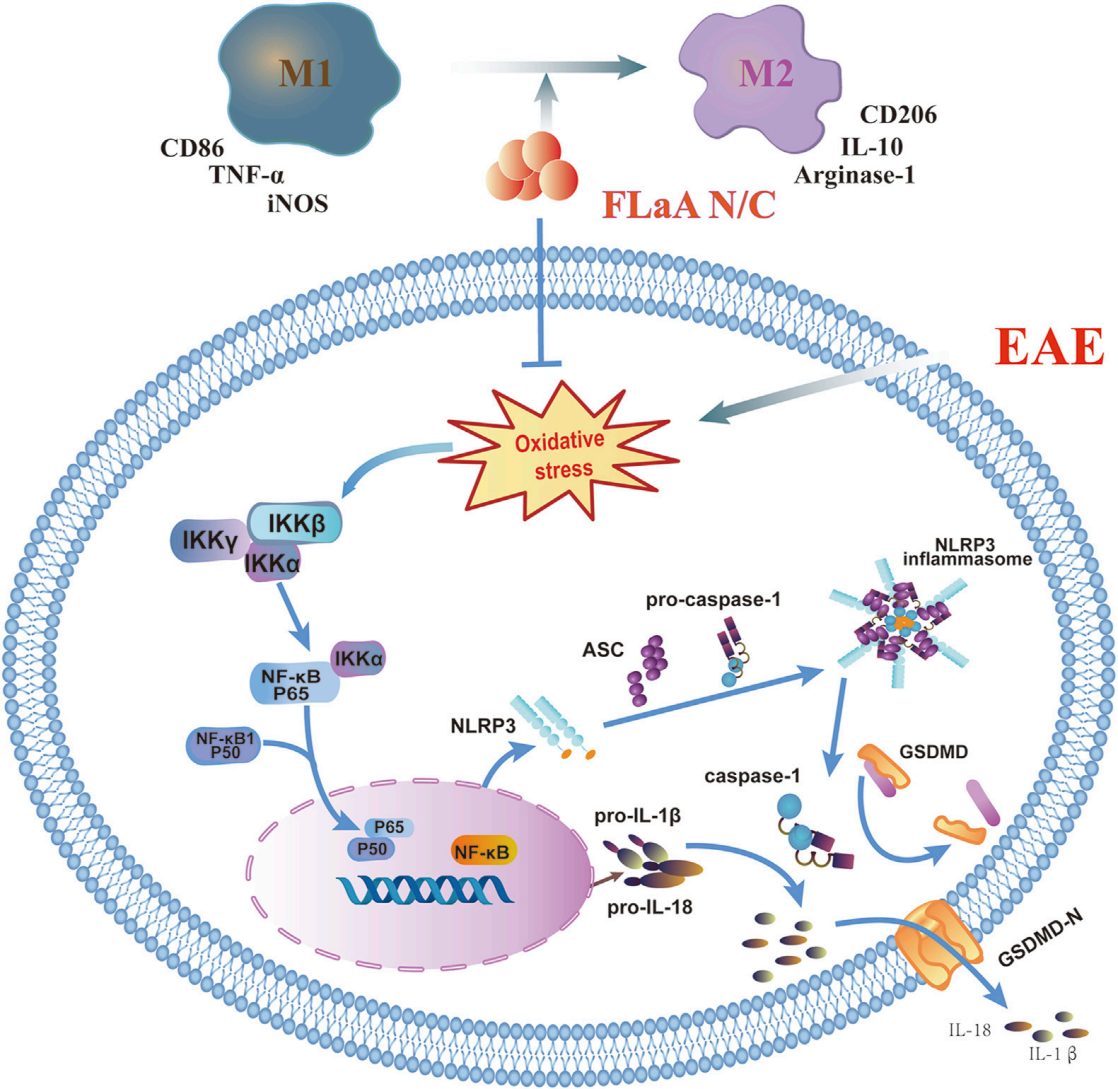
FLaAN/C attenuated inflammatory response by facilitating macrophages/microglia polarization from the M1 phenotype to the M2 phenotype and inhibiting the ROS/NF-κB/NLRP3 signaling pathway, and thus alleviated EAE.

In conclusion, we previously constructed the recombinant FLaAN/C protein to alleviate post-radiation side effects through anti-inflammatory and antioxidant effects. Here, our results showed that FLaAN/C attenuates inflammatory response in mice by facilitating microglia/macrophages polarization from the M1 phenotype to the M2 phenotype and inhibiting the ROS/NF-κB/NLRP3 signaling pathway, and thus alleviates EAE (Figure 8). Our findings provide new evidence of the potential of FLaAN/C as a drug for treating MS.

## Data Availability

The original contributions presented in the study are included in the article/Supplementary Materials, further inquiries can be directed to the corresponding authors.

## Abbreviations

ASC, anti-apoptosis-associated speck-like protein containing a CARD; CASP1, caspase-1 CAT; catalase CD, cluster of differentiation; CNS, central nervous system; COX2, cyclooxygenase 2; DHE, dihydroethidium; EAE, experimental autoimmune encephalomyelitis; GAPDH, glyceraldehyde-3-phosphate dehydrogenase; GSDMD, gasdermin D; IBA1, ionized calcium-binding adapter molecule 1; IFN, interferon; Ig, immunoglobulin; IL, interleukin; iNOS, inducible nitric oxide synthase; LDH, lactate dehydrogenase; LFB, Luxol fast blue stain; MBP, myelin basic protein; MDA, malondialdehyde; MS, multiple sclerosis; MOG, myelin oligodendrocyte glycoprotein; NLRP3, NLR family pyrin domain containing 3; NF, nuclear factor; p, phosphorylated; PBS, phosphate-buffered saline; ROS, reactive oxygen species; SOD, superoxide dismutase; Th, T helper; TNF, tumor necrosis factor; WT, wild-type.
