# Supplementary material for: Novel recombinant protein flagellin A N/C attenuates experimental autoimmune encephalomyelitis by suppressing the ROS/NF-κB/NLRP3 signaling pathway
Source: Front Pharmacol. 2022 Nov 14;13:956402. doi: 10.3389/fphar.2022.956402 (PMC9702353; doi:10.3389/fphar.2022.956402)
Supplement: Supplementary file 1 [file DataSheet10.PDF]

The present study used a novel recombinant protein, namely flagellin A (FlaA) N/C, an independent intellectual property biological agent of our project team. FLaAN/C was developed in an earlier study and is derived from the flagellin protein of *Legionella pneumophila* . The FlaAN/C protein was obtained by our group after constructing plasmids through genetic recombination, sequencing to verify the sequence, expressing the fusion protein, and purifying the protein. At present, the invention patent has been granted (**ZL 2014 10075339.8**)

1.The gene sequence of FlaAN/C in this experiment is as follows:
ATTAACACCAACGTGGCGAGCCTGACCGCGCAGCGTAACCTGGGCGTGAGCGGCAAC
ATGATGCAGACCAGCATTCAGCGTCTGAGCAGCGGCCTGCGTATTAACAGCGCGAAAG
ATGATGCGGCGGGCCTGGCGATTAGCCAGCGTATGACCGCGCAGATTCGTGGCATGAA
CCAGGCGGTGCGTAACGCGAACGATGGCATTAGCCTGGCGCAGGTGGCGGAAGGCGC
GATGCAGGAAACCACCAACATTCTGCAGCGTATGCGTGAACTGAGCGTGCAGGCGGC
GAACAGCACCAACAACAGCAGCGATCGTAGCAGCATTCAGAGCGAAATTAGCCAGCT
GAAAAGCGAACTGGAACGTATTGCGCAGAACACCGAATTTAACGGCCAGCGTATTCTG
GATGGCAGCTTTAGCGGTGGTGGTGGTAGCGGCGGCGGCGGCAGCATTAAACGTATTG
ATGCGGCGCTGAACAGCGTGAACAGCAACCGTGCGAACATGGGCGCGCTGCAGAACC
GTTTTGAAAGCACCATTGCGAACCTGCAGAACGTGAGCGATAACCTGAGCGCGGCGC
GTAGCCGTATTCAGGATGCGGATTATGCGGCGGAAATGGCGAGCCTGACCAAAAACCA
GATTCTGCAGCAGGCGGGCACCGCGATGCTGGCGCAGGCGAACAGCCTGCCGCAGAG
CGTGCTGAGCCTGCTG

2.The fusion plasmid was constructed by DNA recombination technology (enzyme digestion, ligation, transformation, screening and other steps). After sequencing and verifying the sequence, the FLaAN/C protein was induced to be expressed by IPTG. The results are shown in Figure 1

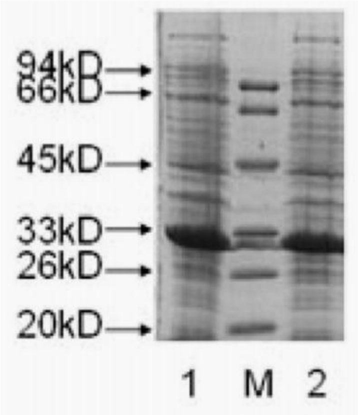

Figure 1: The expression map of FlaAN/C fusion protein (30KD)

3. FlaAN/C fusion protein expression assay and the results are shown in Figure 2

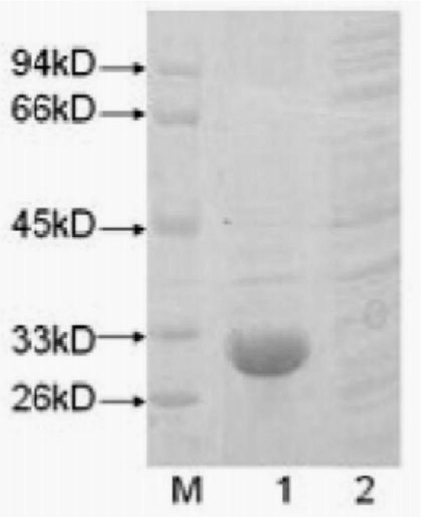

Figure 2: The expression gel map of FlaAN/C

4. The purification results of the FlaAN/C fusion protein (denaturation and purification of inclusion body proteins, and protein denaturation) are shown in Figure 3

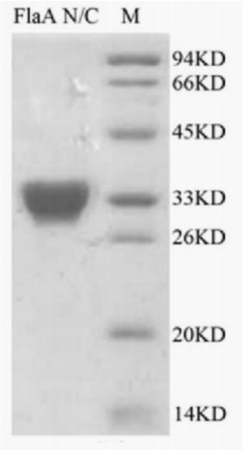

Figure 3: The electrophoresis profile of FlaAN/C purified protein

5. The amino acid sequence of FlaAN/C in this experiment is as follows:

Ile Asn Thr Asn Val Ala Ser Leu Thr Ala Gln Arg Asn Leu Gly Val Ser Gly Asn Met
1 5 10 15 20
Met GIn Thr Ser Ile Gln Arg Leu Ser Ser Gly Leu Arg Ile Asn Ser Ala Lys Asp Asp
25 30 35 40
Ala Ala Gly Leu Ala Ile Ser GIn Arg Met Thr Ala Gln Ile Arg Gly Met Asn Gln Ala
45 50 55 60
Val Arg Asn Ala Asn Asp Gly Ile Ser Leu Ala Gln Val Ala Glu Gly Ala Met GIn Glu
65 70 75 80
Thr Thr Asn Ile Leu GIn Arg Met Arg Glu Leu Ser Val Gln Ala Ala Asn Ser Thr Asn
85 90 95 100
Asn Ser Ser Asp Arg Ser Ser Ile GIn Ser Glu Ile Ser Gln Leu Lys Ser Glu Leu Glu
105 110 115 120

Arg Ile Ala Gln Asn Thr Glu Phe Asn Gly GIn Arg Ile Leu Asp Gly Ser Phe Ser Gly
125 130 135 140

Gly Gly Gly Ser Gly Gly Gly Gly Ser Ile Lys Arg Ile Asp Ala Ala Leu Asn Ser Val
145 150 155 160

Asn Ser Asn Arg Ala Asn Met Gly Ala Leu Gln Asn Arg Phe Glu Ser Thr Ile Ala Asn
165 170 175 180

Leu Gln Asn Val Ser Asp Asn Leu Ser Ala AIa Arg Ser Arg Ile GIn Asp Ala Asp Tyr
185 190 195 200

Ala Ala Glu Met Ala Ser Leu Thr Lys Asn G1n I1e Leu GIn GIn Ala G1y Thr Ala Met
205 210 215 220

Leu Ala GIn Ala Asn Ser Leu Pro Gln Ser Val Leu Ser Leu Leu
225 230 235
